# Vaginal colonisation by probiotic lactobacilli and clinical outcome in women conventionally treated for bacterial vaginosis and yeast infection

**DOI:** 10.1186/s12879-015-0971-3

**Published:** 2015-07-03

**Authors:** Sonal Pendharkar, Erik Brandsborg, Lennart Hammarström, Harold Marcotte, Per-Göran Larsson

**Affiliations:** Division of Clinical Immunology, Department of Laboratory Medicine, Karolinska Institutet at Karolinska University Hospital Huddinge, Stockholm, Sweden; Bifodan AS, Hundested, Denmark; Department of Obstetrics and Gynaecology Kärnsjukhuset, Skaraborg hospital and University of Skövde, Skövde, Sweden

**Keywords:** Bacterial vaginosis, Vulvovaginal candidiasis, Probiotic, Lactobacilli, Metronidazole, Clindamycin, Fluconazole

## Abstract

**Background:**

The aim of this study was to investigate the colonisation by lactobacilli and clinical outcome in women with bacterial vaginosis (BV) and recurrent vulvovaginal candidiasis (R-VVC) receiving antibiotic or anti-fungal treatment in combination with the probiotic EcoVag® capsules.

**Methods:**

A total of 40 Scandinavian women diagnosed with BV or VVC on the basis of Amsel’s criteria or clinical symptoms were consecutively recruited in two pilot open label clinical trials. In trial I, women with BV were treated with clindamycin and metronidazole followed by vaginal EcoVag® capsules, containing *Lactobacillus rhamnosus* DSM 14870 and *Lactobacillus gasseri* DSM 14869, for 5 consecutive days after each antibiotic treatment. In trial II, women were recruited in three groups as follows: women with BV receiving clindamycin and metronidazole treatment together with a prolonged administration of EcoVag® (10 consecutive days after each antibiotic treatment followed by weekly administration of capsules for next four months), women with R-VVC receiving extended fluconazole and EcoVag® treatment, and women receiving extended fluconazole treatments only. The difference in frequency of isolation of EcoVag® strains or other lactobacilli between groups was compared by Fisher’s exact test.

**Results:**

The 6-month cure rate for BV was 50 % in trial I while both the 6- and 12-month cure rates were 67 % in trial II. The 6- and 12-month cure rates for VVC were 100 % and 89 % in women receiving fluconazole and EcoVag®, and 100 % and 70 % in women receiving fluconazole only. The frequency of isolation of any *Lactobacillus* species during the course of the study was associated with cure of BV in trial I and II, whereas the frequency of isolation of EcoVag® strains was significantly associated with the cure of BV in trial II only. As previously observed, a change in sexual partner was associated with relapse of BV with an Odds ratio of 77 (95 % CI: 2.665 to 2225).

**Conclusions:**

The study suggests that the treatment with antibiotics or anti-fungal medication in combination with EcoVag® capsules provide long-term cure against BV and R-VVC as compared to previous reports.

**Trial registration:**

ClinicalTrials.gov NCT02295579. Registered November 20, 2014

**Electronic supplementary material:**

The online version of this article (doi:10.1186/s12879-015-0971-3) contains supplementary material, which is available to authorized users.

## Background

Bacterial vaginosis (BV) and yeast infection are two forms of vaginitis that are common in women. BV is a clinical condition associated with the loss or reduction of colonising *Lactobacillus* species and replacement by a mixed microbiota dominated by *Gardnerella vaginalis* and anaerobic bacteria including *Atopobium vaginae*, *Prevotella* spp.*, Mobiluncus* spp.*, Porphyromonas* spp. and *Peptostreptococcus* spp [1]. It is one of the most common vaginitis affecting women in their reproductive age and symptoms include a malodorous vaginal discharge, itching and pain. The current treatment regimens for BV prescribes vaginal clindamycin or oral/vaginal metronidazole or tinidazole [2, 3]. However, the prescribed doses do not reduce the number of relapses significantly [4–6].

Vaginal yeast infections, the second most common type of vaginitis, is caused due to the fungus *Candida albicans* and is characterised by itching, pain and discharge [7]. It is estimated that 75 % of women will have vulvovaginal candidiasis (VVC) at least once in their lifetime [8]. Equally important is the fact that 40 % to 50 % of subjects have one or more recurrences after the apparent resolution of the first infective episode [9, 10]. A one-time dose of fluconazole is 90 % effective in treating vaginal candidiasis but the treatment is ineffective in cases of recurrent infections [11]. Bacterial vaginosis and yeast infection are associated with increased risk of heterosexual HIV transmission reflecting the need of treating these diseases particularly in sub-Saharan Africa with generalized HIV epidemics where the prevalence of BV and yeast infection is high [12, 13].

Lactobacilli present in a healthy vagina are part of normal bacterial microbiota and protect the host from urogenital infections by maintaining a low pH (<4.5), by producing bacteriostatic and bactericidal substances and through competitive exclusion [14, 15]. The predominant vaginal *Lactobacillus* species are *L. crispatus, L. gasseri, L. iners, L. vaginalis* and *L. jensenii*, with *L. crispatus* being the predominant species in a normal microbiota, whereas *L. iners* although associated with BV, is also prevalent in normal microbiota [16, 17]. In order to reduce the prevalence of BV and yeast infection, a normal vaginal microbiota has to be maintained and the use of probiotic vaginal lactobacilli could serve that purpose. Furthermore, colonising probiotics could be engineered for delivery of microbicide in vagina combining both the specificity of the microbicide with the probiotic activity of the lactobacilli [18].

Several clinical trials have been performed to investigate whether specific strains of lactobacilli, administered intra-vaginally or orally, in combination with antibiotics or not, are able to colonise the vagina in women with bacterial vaginosis and yeast infection and improve symptoms [19–22]. We have recently performed a study showing that an aggressive antibiotic treatment along with vaginal *Lactobacillus* administration could provide a long lasting cure [22]. Women were given a seven days course of daily 2 % vaginal clindamycin cream together with 300 mg of oral clindamycin and vaginal metronidazole gel. Oral and vaginal capsules containing different vaginal strains of lactobacilli both newly characterised and commercial ones were given for 5 days after each antibiotic course. Oral clindamycin was also given to their sexual partners. The cure rate was 74.6 % after 6 months, 65.1 % after 12 months and 55.6 % after 24 months. The experiment showed that the lactobacilli contained in EcoVag® vaginal capsules (a mixture of *L. rhamnosus* DSM 14870 and *L. gasseri* DSM 14869) were the best colonisers but no correlation was found between colonisation and cure of BV.

In this paper we investigated the efficacy of a combination of antibiotics and EcoVag® for treatment of BV and tested if colonisation and treatment efficacy of *L. gasseri* DSM 14869 and *L. rhamnosus* DSM 14870 could be improved by increasing the dose frequency of EcoVag® capsules. We also tested for the first time, a combination of EcoVag® and an anti-fungal drug for treatment of recurrent yeast infection and evaluated if *L. rhamnosus* DSM 14870 and *L. gasseri* DSM 14869 can colonise women when the microbiota is not disturbed by antibiotics.

## Methods

### Lactobacilli used for treatment

Commercially available EcoVag® *Lactobacillus* strains which were successfully colonising in our previous study were chosen for the clinical trials [22]. EcoVag® capsules (Bifodan A/S, Denmark) contain *L. gasseri* (DSM 14869) and *L. rhamnosus* (DSM 14870) at  1× 10^8^ CFU of each strain/capsule.

### Clinical Study

Two pilot open label follow-up clinical trials were performed. The first clinical trial including patients with BV was conducted in an outpatient private gynaecological clinic in Drammen, Norway from July 2009 until January 2011 and the second trial, including both patients with BV and yeast infection, was carried out in Skövde or Örobro, Sweden from September 2011 until October 2014. In the first trial, patients with history of BV that visit the clinic in Drammen were invited to participate in the study. In trial II, patients with BV or VVC visiting clinics in Västra Götaland County, Sweden were asked to participate in the study at the clinic in Skövde. The patients were consecutively added to the studies. Women included in the study were regularly menstruating, 18 years or older, with normal gynaecological status, not pregnant or breast-feeding and without signs of other genital tract infections. Exclusion criteria were patients with hormonal IUD without regular menstruation; women infected with *Chlamydia trachomatis* or *Trichomonas vaginalis*.

### Study sample

A total of 10 women in the first and 30 women in the second clinical trial were included. The mean age was 35.7 years with a range of 26–49 years in the first trial and 32 with a range of 22–43 years in the second trial.

### Clinical method and diagnosis of BV and candidiasis

At inclusion, women had a routine gynaecological examination with a non-lubricated speculum and a vaginal ultrasound. A sample of vaginal secretion was analyzed for vaginal pH using special pH strips (range 3.8-5.0). The diagnosis of BV was based on the Amsel criteria [23], ie. fulfilling at least three of four criteria; thin homogenous discharge, vaginal pH above 4.5, positive amine test and presence of clue cells during microscopic investigation using a phase contrast microscope.

Yeast infection was diagnosed on the basis of clinical examination confirming vaginal discharge, a wet smear and a KOH smear with the presence of alkali resistant blastospores or hyphae of *C. albicans* observed using a phase contrast microscope (at magnification of 400 times). Vaginal samples were also tested to exclude *C. trachomatis* infection using strand-displacement amplification (CT amplified DNA assay; Becton-Dickinson, New Jersey, USA) according to the local laboratory routine.

### Treatment

After signing the informed consent form, women were given the antibiotic or anti-fungal treatment followed by EcoVag® vaginal capsules. Treatment was given as follows:

### Trial I: Treatment of BV

**Fig. 1:**
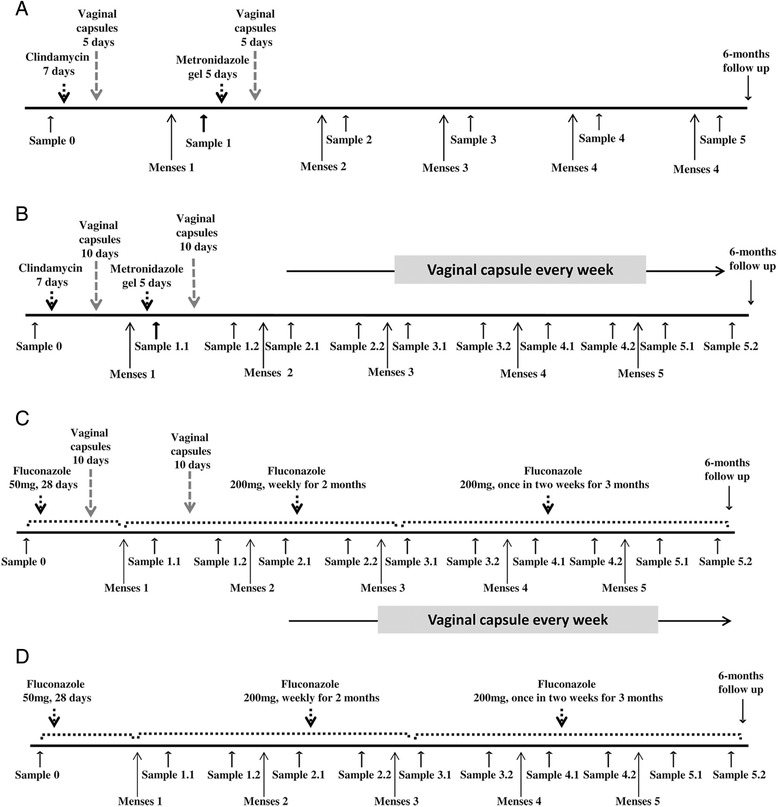
Time schedule of the treatment of BV and VVC and follow up. **a**) Trial I, women treated for BV with antibiotics and EcoVag®, **b**) Trial II, women treated for BV with antibiotics and prolonged administration of EcoVag®, **c**) Trial II, women treated for VVC with fluconazole and prolonged administration of EcoVag® and **d**) Trial II, women treated for VVC with fluconazole only

The first clinical trial was designed with one group of 10 women diagnosed with BV. The treatment given was similar to our previous study [22] except for the metronidazole course which was given only once instead of twice. Women in the BV group were given a seven day course of daily 2 % vaginal clindamycin cream (Dalacin vaginal cream 2 %, Pfizer Norway Ltd) together with oral clindamycin 300 mg BID for 7 days (Dalacin 300 mg, Pfizer Norway Ltd). Directly after the clindamycin treatment, a new treatment was started with EcoVag® capsules for 5 days. After the next menstruation, women were given a 5 days course of vaginal 0.75 % metronidazole gel (Zidoval gel 75 g, MedaAS, Norway) followed by 5 more days with EcoVag® (Fig. 1a). Oral clindamycin treatment was also given to the patients sexual partners [24]. The efficacy of treatment was evaluated after the last treatment course. Since a placebo group was included in a previous study [25], we did not include this group in the present trial for ethical reasons.

### Trial II: Treatment of BV and yeast infection with increased dose frequency of EcoVag®

In the second trial, women were recruited in the three groups as follows; group-1: 11 women with BV receiving antibiotics and EcoVag®, group-2: 9 women with recurrent vulvovaginal candidiasis (R-VVC) receiving anti-fungal drug and EcoVag® and group-3: 10 women with *Candida* infection receiving only the anti-fungal drug. Women in group-1 received a similar antibiotic treatment for BV as in the first trial and a prolonged treatment with EcoVag®. After each antibiotic course, EcoVag® capsules were given for 10 days and after the second menstruation once every week for the next four months (Fig. 1b). Patient’s sexual partners were also treated with oral clindamycin.

In group-2, women with *Candida* infection were treated with anti-fungal medication and EcoVag® capsules. They were given 28 days course of fluconazole 50 mg every day and vaginal EcoVag® capsules for 10 days from day 18 to 28 (Fig. 1c). After the first menstruation, EcoVag® capsules were given again for 10 days along with a weekly course of 200 mg fluconazole for two months. This was followed by a third course of fluconazole, where women were given 200 mg of the drug once every two weeks for the next three months. After the second menstruation, EcoVag® capsules were given once every week for four months.

Women with candidiasis in group-3 received a similar anti-fungal treatment but no EcoVag® capsules (Fig 1d).

### Follow up

The primary outcome measure of the studies was cure of BV and VVC while the secondary outcome measure was colonisation by EcoVag® *Lactobacillus* strains and other lactobacilli. In the first trial, after every menstruation women took a self-swabbed vaginal culture and a glass smear that was air dried and sent by regular mail to our laboratory at the division of Clinical Immunology, Karolinska University Hospital, Huddinge. Samples were sent in the provided ready to use envelopes. Similarly samples were collected in the second trial but twice a month. After every menstruation, women took the self-swabbed sample and glass smear at days 7 and 21.

Every woman was followed up by a phone call from the investigation nurse and asked about the treatment complications and concomitant medication. If the patient had failed to send a sample, it was recorded as missing but she was reminded and asked to send in the next sample. Each patient collected a total of 6 or 10 vaginal samples in first and second trial respectively. After six menstrual cycles, women were scheduled for a follow up visit to the clinic. In the second trial, the women in group-1 and group-2 were then followed up to 12 months. Women in the BV groups were considered cured at the 6- and 12-months follow up if they had none of Amsel’s critera fulfilled. The women in the VVC group were considered cured on the absence of vaginal discharge and absence of alkali resistant blastospores or hyphae of *C. albicans* in the wet and KOH smears.

### Cultivation of lactobacilli

Upon arrival at the laboratory, vaginal swabs were directly streaked onto three of each Rogosa agar (BD Difco™ Rogosa SL agar, Becton, Dickinson and Company, Spark, MD) and Columbia agar (BD Difco™ Columbia Blood Agar Base) with 5 % horse blood. Plates were incubated for 48 hours at 37 °C in anaerobic condition using BD GasPack™ EZ gaz generating systems (Becton, Dickinson and Company). Colonies with *Lactobacillus* morphology and yielding bacilli were re-streaked. Respective isolated colonies from re-streaked plates were used to inoculate MRS broth medium. Tiny transparent colonies (*L. iners* like) from blood agar plates yielding Gram-positive bacilli were directly collected from the plates and were used for genomic DNA isolation. Glycerol stocks (15 %) were prepared and stored at −80 °C. Four to eight colonies were picked per sample.

### Identification of the EcoVag® Lactobacillus strains

Genomic DNA was extracted from lactobacilli using Qiagen’s DNAeasy Blood & Tissue extraction kit (Qiagen GmbH, Hilden, Germany) and Invisorb Universal Bacteria HTS 96/V (STRATEC Molecular GmbH, Berlin, Germany) and the strains were typed using PCR amplification on the bacterial repetitive extragenic palindromic DNA sequences (REP-PCR). The primers REP1 (5’-IIIICGICGICATCIGGC-3’) and REP2 (5’-ICGICTTATCIGGCCTAC-3’) were used on the basis of reproducibility, band intensity and discriminative power as described previously [26]. The DNA profiles were compared with those of EcoVag® strains [22]. If the profiles matched, then the identification was confirmed by using RAPD (Random amplified polymorphic DNA) PCR with primers for both the *Lactobacillus* species.

Identification of the isolates was confirmed by using one of the primers for each EcoVag® strain: RAPD4 (5’-CCGCAGCCAA-3’) (primer 1254, [27]) and RAPD6 (5’-TGGGCGTCAA-3’) (primer OPL-2, [28]) for *L. gasseri* DSM 14869 and RAPD1 (5’-ATGTAACGCC-3’) (primer P2, [29]) and RAPD4 for *L. rhamnosus* DSM 14870. When it was difficult to confirm the DNA profiles using these primers, a third primer, RAPD17 (5’-AACGCGCAAC-3’) [30] was used to confirm the identification. Primers were used at 0.5 μM employing the same PCR mixture as described for the REP-PCR as previously described [22].

### Statistical analysis

The age and length of symptoms in BV and VVC groups were compared by Mann–Whitney U test. The frequency of isolation was determined as the percentage of samples positive for EcoVag® strains or other lactobacilli on the total number of samples in each group. The difference in frequency of isolation of EcoVag® strains or other lactobacilli between groups was compared by Fisher’s exact test. Two-tailed P values less than 0.05 were considered statistically significant. Odds ratio (OR) to analyze association between change of partner and relapse of BV was calculated at 95 % confidence interval (CI) using contingency table analysis. All the comparisons were performed with GraphPad Prism 4 software (GraphPad Software, Inc., La Jolla, Ca) and in accordance the intention to treat (ITT) principle.

### Ethical approvals and registration

The trials were performed in accordance with the Declaration of Helsinki*.* The trial I was approved by the southeast regional Ethics Committee in Oslo, Norway and the trial II by the regional Ethics Committee in Göteborg, Sweden. The clinical trials were registered on November 20, 2014 at ClinicalTrials.gov with identification number NCT02295579 (http://clinicaltrials.gov/show/NCT02295579).

## Results

### Demographic data

A total of 10 women with BV were recruited in trial I. Out of 30 women recruited in trial II, two women dropped out from the study. Hence, there were 9 women in group-1 (BV), 9 in group-2 (R-VVC) and 10 women in group-3 (R-VVC control group) (Additional file 1: Figure S1). The demographic data for 38 women in trial I and trial II, who completed the study until the six-month follow up is shown in Table 1. Among the 17 women with BV in trials I and II, 14 had a history of BV. Mean length of symptoms of BV was 14 months with a range of 1 to 36 months. All the women with yeast infection had a history of VVC with a mean of 42 months of having symptoms (range 12–120 months).

**Table 1:** Demographics and clinical characteristics of the study groups at enrollment.^a^ Each group received EcoVag® capsules containing the mix of *L. gasseri* DSM 14869 and *L. rhamnosus* DSM 14870

Groups^a^	Trial I	Trial II		
	BV EcoVag®	Group-1 BV EcoVag®	Group-2 VVC EcoVag®	Group-3 VVC No EcoVag®
	n = 10	n = 9	n = 9	n = 10
**Mean age (range)**	35.7 (26–49)	30.0 (19–43)	33.4 (23–43)	32.5 (22–43)
**Patients with history of BV/VVC (no.)**	6	8	9	10
**Length of symptoms (months, mean and range)**	11 (0.5-30)	17.5 (1–36)	46.6 (12–120)	37.2 (12–120)

n = number of women who completed the study with 6-month follow up

### Colonisation with EcoVag® strains

#### First trial: Treatment of BV

**Fig. 2:**
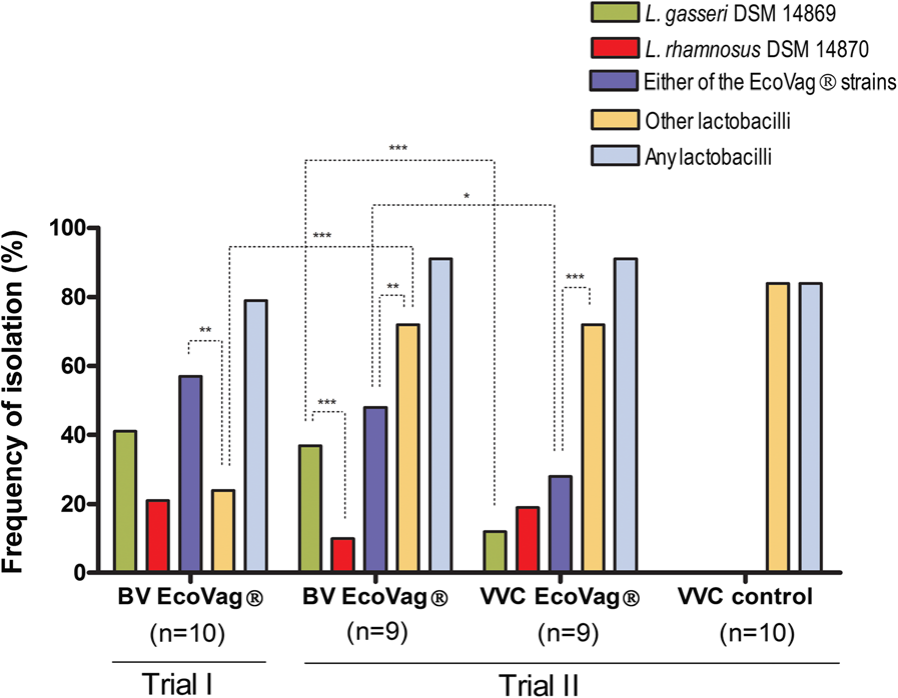
Frequency of isolation of EcoVag® and other *Lactobacillus* strains during the treatment of BV and VVC. The frequency of isolation (y axis) was determined as the percentage of samples positive for EcoVag® strains or other lactobacilli on the total number of samples for each group. Trial I: women treated for BV with antibiotics and EcoVag® (BV EcoVag®). Trial II: women treated for BV with antibiotics and prolonged administration of EcoVag® (BV EcoVag®), women treated for VVC with fluconazole and prolonged administration of EcoVag® (VVC EcoVag®), women treated for VVC with fluconazole only (VVC fluconazole). The difference in frequency of isolation of EcoVag® strains or other lactobacilli between groups was compared by Fisher’s exact test. *** P < 0.001

None of the women were colonised by any lactobacilli before administration of EcoVag® capsules. Following administration, nine out of ten women were colonised by either of the EcoVag® *Lactobacillus* strains during the study (Fig. 2, Additional file 2: Figure S2; Additional file 3: Table S1,). In eight women, EcoVag® strains persisted for at least two weeks after stopping the treatment (Additional file 2: Figure S2). In three women (no. 2, 6 and 9), either of the strains was identified at month 4 (two months after the treatment was stopped) and persisted until month five (three months aft-er the treatment) in two of them (no. 6 and 9). *L. gasseri* DSM 14869 was more frequently isolated than *L. rhamnosus* DSM 14870 (17 vs 9 out of 42 samples) but the difference was not significant (P = 0.098) (Additional file 3: Table S1).

#### Second trial: Treatment of BV and yeast infection with long-term administration of EcoVag®

In the second trial, we evaluated if increasing the EcoVag® dose frequency would improve colonisation with *L. rhamnosus* DSM 14870 and *L. gasseri* DSM 14869 in BV patients and if the administered EcoVag® strains could colonise yeast infected patients, ie. when the microbiota has not been disturbed with antibiotics. We also increased the number of samples collected from one to two between each menstruation as the level of lactobacilli may vary during the menstruation cycle. Sample 1 was taken after menstruation (day 7) and immediately before the first weekly EcoVag® treatment.

In group-1 with BV positive women, seven out of nine women regularly sent the samples until month six except for woman no. 13 and 18 (Additional file 2: Figure S2). In five of these women (no. 4, 14, 15, 17 and 20) either of the EcoVag® strains was identified until month five. Overall, *L. gasseri* DSM 14869 was isolated more frequently (32 out of 86 samples, 37 %) than *L. rhamnosus* DSM 14870 (9 out of 86 samples, 10 %) (P < 0.001) (Fig. 2, Additional file 3: Table S1).

Non-EcoVag® lactobacilli were in majority identified in 63 out of 86 samples (73 %) from 9 women compared to EcoVag® strains identified in 41 samples (48 %) (P < 0.002) (Fig. 2, Additional file 3: Table S1). Interestingly, four women (no. 2, 10, 14 and 18) diagnosed with BV had lactobacilli at the time of recruitment and EcoVag® strains were more frequently isolated from women that did not have lactobacilli at the start of the study (29 out of 48 samples (60 %) from 5 women) compared to women who had lactobacilli at the start of the treatment (12 out of 38 samples (31.5 %) from four women) (P < 0.01) (Additional file 2: Figure S2). No significant difference was observed in the frequency of isolation of EcoVag® strains or other lactobacilli between sample 1 (day 7) and sample 2 (day 21).

A comparison of both trials I and II shows that colonisation by EcoVag® lactobacilli in women with BV did not improve significantly upon increasing the dose (Fig. 2). For this comparison, only sample I of trial II was included as it corresponds to the sample taken in trial I. In month 4, EcoVag® strains were isolated in more women in trial II (6 out of 8) than in trial I (3 out of 8) but this was not significant (P = 0.31) (Additional file 2: Fig. S2). Overall, EcoVag® strains were identified in 24 out of 42 (57 %) samples in trial I, and 24 out of 48 (50 %) samples in trial II. The frequency of isolation of other *Lactobacillus* species was significantly higher in the second trial (34 out of 48 samples, 71 %) than in the first trial (10 out of 42 samples, 24 %) (P < 0.001). The frequency of isolation of any lactobacilli (EcoVag® strains or other *Lactobacillus* strains) was similar for trials I and II (33 out of 42, 79 % vs 45 out of 48, 94 %, P = 0.06).

In the *Candida* infected patients (group-2), EcoVag® strains were isolated from 8 out of 9 (89 %) women at some time point during the study but the *Lactobacillus* microbiota was dominated by non EcoVag® *Lactobacillus* strains (Fig. 2, Additional file 2: Figure S2). Overall *L. rhamnosus* DSM 14870 and *L. gasseri* DSM 14869 were isolated in 16 (19 %) and 10 (12 %) out of 86 samples respectively (Fig. 2, Additional file 3: Table S1). Furthermore, as observed before, EcoVag® strains were more frequently isolated from women that did not have lactobacilli at the start of the study (15 out of 31 samples (48 %) from 3 women) compared to women from whom lactobacilli were isolated (9 out of 53 samples (17 %) from 6 women) (P < 0.01).

A comparison of group-1 and group-2 in trial II showed that EcoVag® lactobacilli were isolated more frequently in BV patients pretreated with antibiotics (41 out of 86 samples, 48 %) than from *Candida* infected patients pretreated with anti-fungal drugs (24 out of 86 samples, 28 %) (P < 0.05).

In *Candida* infected patients receiving fluconazole only (group-3), lactobacilli were regularly isolated from 9 out of 10 women. Other *Lactobacillus* species isolated in all three groups in trial II were *L. gasseri*, *L. crispatus*, *L. iners, L. jensenii, L. vaginalis*, *L. reuteri*, *paracasei*, *L. plantarum*, *L. rhamnosus*, *L. fermentum*, *L. acidophilus* and *L. salivarius* (Additional file 3: Table S2). *L. gasseri* was the most frequently isolated species in women with BV receiving EcoVag® while *L. crispatus and L. gasseri* were the most prevalent species in women with VVC receiving EcoVag® (group-2) or fluconazole only (group-3). A significantly higher proportion of samples positive for *L. crispatus* were found in women with *C. albicans* infection (group-2 and group-3) (Additional file 3: Table S3) but a higher proportion of women in group-2 and group-3 were already positive for *L. crispatus* at the beginning of the study.

### Clinical Outcome

#### First trial: Treatment of BV

**Fig. 3:**
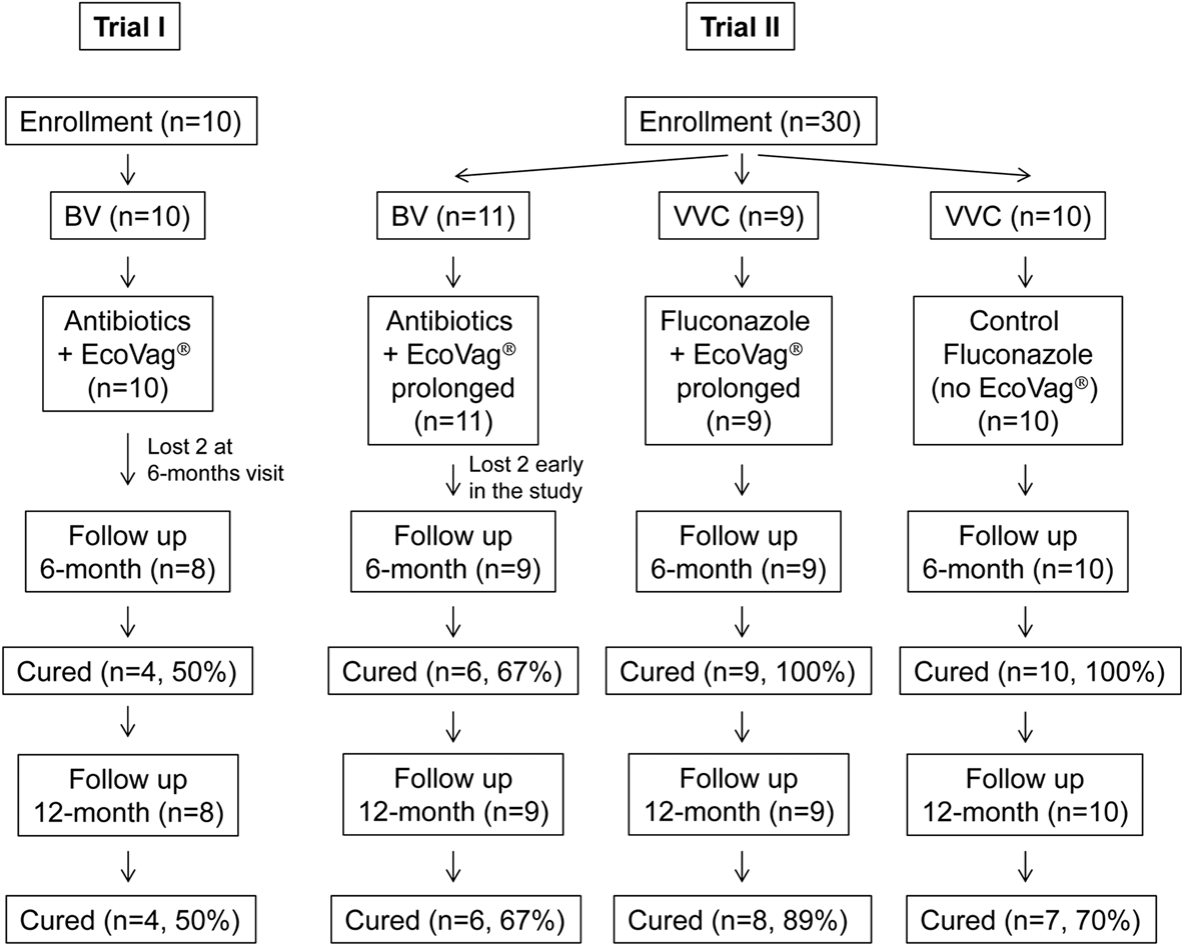
Cure rate of BV and VVC following different treatments. Two women in Trial I and three women in Trial II group-1 had a new sexual partner during the study and developed symptoms and experienced relapse of BV

Among the 10 women in this group, two women did not visit the clinic for the 6 month follow up and were therefore excluded from the analysis on clinical outcome. The cure rate after month 6 was 50 % with four women cured and four with a relapse of BV (Table 2, Fig. 3).

**Table 2:** Clinical outcome

Groups	Trial I	Trial II		
	BV EcoVag®	Group-1 BV EcoVag®	Group-2 VVC EcoVag®	Group-3 VVC No EcoVag®
Patients enrolled	10	11	9	10
Patients lost to follow up	2 (no. 5 and 20)^a^	2 (no. 12, 19)	0	0
Patients analyzed	8	9	9	10
Patients cured at 6-months	4 (50 %)	6 (67 %)	9 (100 %)	10 (100 %)
Patients cured at 12-months	4 (50 %)	6 (67 %)	8 (89 %)	7 (70 %)
Relapses (identification number)	4 (no. 1, 2, 7, 8)	3 (no. 2, 4, 17)	0	3 (no. 23, 25, 28)
Patients with new sexual partner on follow up	2 (no. 2, 7)^b^	3 (no. 2, 4, 17)	1 (no. 7)	0
New sexual partner before relapses of BV	2 (no. 2, 7)	3 (no. 2, 4, 17)	0	0
*Chlamydia* infection	0	1 (no. 4)	0	0
Surgical abortion and post-operative infection	0	1 (no. 17)	0	0
Both BV and VVC	0	1 (no. 15)^c^	0	0

^a^Number of patients and identification number in parenthesis

^b^Data not available for one woman (no. 1)

^c^The patient got cured of BV

**Fig. 4:**
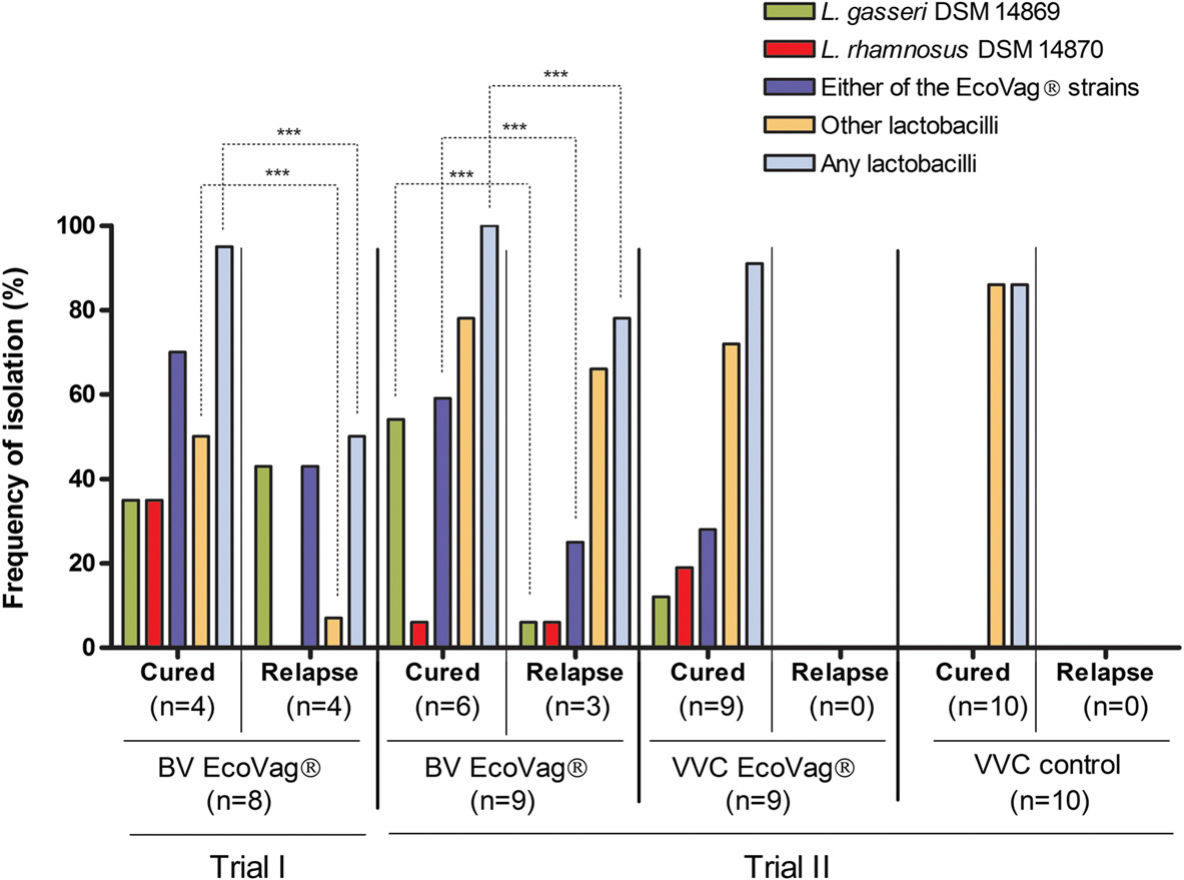
Association between the frequency of isolation of EcoVag® and other *Lactobacillus* strains with cure of BV and VVC (until 6-month follow up). The frequency of isolation (y axis) is determined as the percentage of samples positive for EcoVag® strains and other lactobacilli on the total number of samples for each group (cured or relapse). Trial I: women treated for BV with antibiotics and EcoVag® (BV EcoVag®). Trial II: women treated for BV with antibiotics and prolonged administration of EcoVag® (BV EcoVag®), women treated for VVC with fluconazole and prolonged administration of EcoVag® (VVC EcoVag®), women treated for VVC with fluconazole only (VVC fluconazole). The difference in frequency of isolation of EcoVag® strains or other lactobacilli between cured women and woman with relapse (until 6-month follow up) was compared by Fisher’s exact test. *P < 0.05, **P < 0.01, ***P < 0.001

The frequency of isolation of EcoVag® strains was slightly higher in cured women (14 out of 20 samples) than in women with relapse (6 out of 14 samples) but was not significant (P = 0.16) (Fig. 4, Additional file 3: Table S4). However, the frequency of isolation of any lactobacilli was associated with the cure of BV (Fig. 4, Additional file 3: Table S4). Nineteen out of 20 (95 %) samples from the four cured women contained either EcoVag® strains or other *Lactobacillus* strains compared to only 6 samples out of 14 (43 %) from women who experienced a relapse (P < 0.01).

#### Second trial: Treatment of BV and yeast infection

Nine women in group-1 completed the study and the cure rate for BV after month 6 and 12 was 67 % with 3 relapses and 6 cured women (Table 2, Fig. 3). An association was found between the cure of BV and both the presence of EcoVag® strains or any lactobacilli. The frequency of isolation of EcoVag® strains was higher in cured women (33/54 samples, 61 %) than in women with relapse (8/32 samples, 25 %) (P < 0.01) (Fig. 4, Additional file 3: Table S4). Furthermore, all the 54 samples (100 %) from cured women were identified with lactobacilli compared to 25 out of 32 (78 %) samples in women with relapse (P < 0.001).

Three women who had new sexual partner during the study developed symptoms and experienced relapse while the others (six women) with their same partners remained cured (P < 0.05). Overall in both trials, among 17 women treated for BV, 5 women changed their partners during the follow up and experienced relapse while only one woman that did not change partner had a relapse. Thus, the change of sexual partner was associated with the relapse of BV, OR 77 (2.665 to 2225 at 95 % CI, P = 0.0014).

All nine women infected with *Candida* and treated with fluconazole and EcoVag® (group-2) were cured at the 6-month follow up (Table 2, Fig. 3). All the treated women in this group visited the clinic for a second follow up (12–18 months after the initial treatment) and eight were still cured (89 % cure rate).

In the group receiving fluconazole only (group-3), all the women were also cured at the 6-month follow up but three women got a relapse before 12-months follow up and were given a new course of fluconazole treatment plus EcoVag® (Table 2, Fig. 3). The 12-month cure rate was thus slightly higher in women receiving fluconazole and EcoVag® (89 %) than in women receiving fluconazole only (70 %) but this was not statistically significant (P = 0.582). No association was found between cure of VVC and isolation of EcoVag® strains or other lactobacilli.

### Adverse events

Only a few adverse events were reported during the intervention period in both trials. In trial I, one man treated with clindamycin had a skin rash and stopped taking the antibiotic after six days. In trial II, women 6 and 9 in group-2 stopped taking the last 10 and 2 capsules respectively.

## Discussion

Various approaches for the treatment of BV have been applied but recurrence has remained a major challenge. We previously showed that supplement therapy with EcoVag® capsules could improve the efficacy of antibiotics for treatment of BV [25]. The cure rate at 6-month improved from 46 % to 65 % in women receiving EcoVag®, a nearly 20 % difference. In a more recent study [22], an aggressive antibiotic treatment combined with lactobacilli gave a cure rate of 75 % and 65 % after 6 and 12 months respectively. In this study we further tried to understand and improve colonisation with EcoVag® *Lactobacillus* strains and cure rate of BV and R-VVC.

In trial I, the treatment with antibiotics and dose of EcoVag® given to the women was similar to our previous study [22] except for the metronidazole course which was given once instead of twice as it might affect the colonisation by lactobacilli. It was previously shown that lactobacilli are sensitive to high concentration of metronidazole [31]. Either of the EcoVag® strains, *L. rhamnosus* DSM 14870 or *L. gasseri* DSM 14869, colonised 90 % of the women and persisted up to 3 months after stopping the treatment in 25 % of the treated women. These results are slightly better than our previous study where EcoVag® strains were isolated in 75 % of the women and in 13 % of them, three month after stopping the treatment [22]. This is mainly due to the fact that colonisation by *L. gasseri* DSM 14869 was higher in the present study*.*

In comparison, other probiotic strains (*L. crispatus* CTV-05, *L. rhamnosus* GR-1 and *L. reuteri* RC-14) were shown to colonise up to 70 % of the women after one month [32–35]. Very few studies have been performed on long term monitoring of *Lactobacillus* colonisation following vaginal administration. Similar to the present study, Ehrström *et al*. administered a mixture of 5 strains of lactobacilli after conventional treatment with clindamycin. They found that 53 and 26 % of the women were colonised by any of the strains after the first and second menstruation following administration of lactobacilli [30]. Nine percent of the women were still colonised 6 months after administration of the lactobacilli.

These results suggest difficulties to maintain long-term colonisation with administered lactobacilli. In a study performed by Eriksson K *et al.,* clindamycin was found to be present in the vagina in lower concentrations 5 days after the treatment was ceased [36]. Considering this, and in order to improve the persistence of EcoVag® strains, we modified our treatment protocol in trial II by increasing the EcoVag® dose to 10 days after antibiotic treatments followed by administration of EcoVag® capsules once weekly for four months.

Colonisation and persistence of EcoVag® lactobacilli was slightly improved by increasing the EcoVag® dose with a higher proportion of women colonised by EcoVag® strains at month 4 (75 % vs 38 %) but this was not significant. Furthermore, the frequency of isolation of EcoVag® strains was similar in both trials. However, the colonisation by other *Lactobacillus* species was significantly higher in the second trial. The prolonged treatment with EcoVag® capsules may favorise colonisation by other lactobacilli by changing the vaginal environment. It might also be because a higher proportion of women harbored other lactobacilli at the time of inclusion in trial II (44 %) than in trial I (0 %) and those women were colonised less efficiently by the EcoVag® *Lactobacillus* strains. Antonio *et al*. has described that colonisation by probiotic *L. crispatus* CTV-05 is decreased in the presence of endogenous lactobacilli [34].

It has previously been shown that the composition of vaginal microbiota changes during menstrual cycle [37, 38]. In order to verify any change in *Lactobacillus* colonisation right after the cessation of bleeding and before the menses, vaginal swab samples and vaginal smears were collected twice a month on day 7 and 21 after menstruation in trial II. No difference was observed between isolation of EcoVag® strains or other lactobacilli over the cycle. These results contrast with studies that report an increase in the proportion of *Lactobacillus* species over the menstrual cycle and that of non-*Lactobacillus* species at menses [37, 38].

The previous *Lactobacillus* colonisation studies suggested that exogenously administered lactobacilli can colonise and persist in the vagina of BV patients pretreated with antibiotics [22]. However, it was not clear how well the given *Lactobacillus* strains would colonise when, the vaginal microbiota has not been previously reduced by antibiotics. To answer this question, we included a group of yeast infected women in trial II who received an aggressive fluconazole treatment with a similar administration of EcoVag® capsules. Overall, EcoVag® strains colonised better in women who were treated for BV with antibiotics than in yeast infected women treated with anti-fungal drugs. This could be due to an inefficient colonisation by EcoVag® lactobacilli in the absence of prior antibiotic treatment. This is also suggested by the fact that EcoVag® strains were more frequently isolated from women with yeast infection that did not have lactobacilli at the start of the study. As stated earlier, the presence of certain lactobacilli may affect colonisation with probiotic lactobacilli [39].

The 6-month cure rate in women treated for BV (50 % and 67 % in trial I and II respectively) is better than in most published studies where the cure rates reported are around 40 % six months after treatment [5, 25, 40, 41] but lower than in our previous one (75 %). This might be because only one course of metronidazole was given in the present study instead of two [22]. The cure rate at month 12 in trial II (67 %) was however comparable to the one previously reported (65 %) [22]. Two studies reported higher cure rate (69 and 91 %) at 12-months in women treated with antibiotic and adjuvant lactobacilli but only women using natural method of contraception were included suggesting that they were probably engaged in a more stable relationship and less prone to change of partners and relapses [21, 42]. In the present study, the 12-month cure rate in trial II would be 100 % if we remove women having new sexual partner during treatment.

In our previous study, we observed no correlation between isolation of EcoVag® strains and cure of BV but we did not consider long term persistence of lactobacilli [22]. In trial II, we observed that the frequency of isolation of EcoVag® over the course of the study was associated with cure of BV. There was also a significant association between frequency of isolation of any lactobacilli and cure of BV in trials I and II. These results suggest that prolonged colonisation by EcoVag® strains or any lactobacilli following the antibiotic treatment might provide a long lasting cure of BV. EcoVag® strains could promote the colonization by a particular *Lactobacillus* species which confer stability of the vaginal microbiota. The isolation of some *Lactobacillus* species like *L. crispatus* have been associated with the absence of BV [16, 43]. In Trial II, women with BV treated with EcoVag® were colonized with various species, *L. gasseri* being the predominant one. Since all three women that got relapse in trial II changed partners during treatment, it is difficult to conclude that there is an association between colonization by a specific species and protection against relapse of BV.

As previously reported, relapse of BV was strongly associated with a change of sexual partner during the follow up [22]. Based on these results it can be stated that change in sexual partner is strongly associated with BV. This could be due to an increased sexual activity after change of partner or because BV may act as a STD [21]. Sexual activity has been shown to affect colonisation by exogenous or endogenous lactobacilli [34, 35].

The few clinical studies performed to date regarding supplementation of lactobacilli after conventional antifungal treatment in women with VVC show conflicting results and it is still controversial whether probiotics can prevent recurrences of VVC [19, 30, 44, 45]. Sobel e*t al.* reported the efficient use of long term oral fluconazole treatment for R-VVC [11] where cure rates in treatment group after 6 and 12 months were 91 % and 43 % compared to 36 % and 22 % in the placebo group. We thus evaluated if supplementation of EcoVag® during long- term oral fluconazole treatment could increase the cure rate. We observed a high cure rate with all the women cured after six months of treatment with anti-fungal supplemented with EcoVag® strains and 89 % still cured after 12 months. Although not significant, a lower 12-months cure rate was observed in women receiving fluconazole but no EcoVag® (70 %). The EcoVag® strains could slightly improve the treatment efficacy by directly inhibiting yeast growth or adherence or by supporting colonisation by pre-existing lactobacilli.

The sample size in this study was rather small and the culture-based method used favors the identification of the lactobacilli dominating the microbiota which might explain why the EcoVag® strains were only sporadically detected for some women. Therefore, our results need to be confirmed in a larger cohort using molecular methods for quantification of the EcoVag® strains. Furthermore, the impact of administration of EcoVag® on the whole vaginal microbiome should also be measured to better understand how the administered EcoVag® strains helps in restoring the normal microbiota and reduce relapse of BV and VVC.

## Conclusion

This pilot study suggests that the treatment with antibiotics or anti-fungal medication in combination with EcoVag® *Lactobacillus* strains provide long-term cure against BV and R-VVC as compared to the previous published studies. We could confirm our previous finding that a change of partner was strongly associated with relapse of BV. Further studies with larger cohorts and using quantitative molecular methods will have to be performed to confirm the relationship between colonisation by EcoVag® *Lactobacillus* strains and cure of BV and VVC.

## Additional files

Additional file 1: Figure S1. Participants flow diagram. Additional file 2: Figure S2. Isolation of EcoVag® strains and other lactobacilli during the treatment. Some samples were not provided during the studies (No sample). Women with a new sexual partner (New partner), cured women (√) and relapse (X) at 6-month follow up are indicated. Additional file 3: Table S1, Table S2, Table S3 and Table S4. Colonisation with lactobacilli: number of women colonised and frequency of isolation. Table S2 Identification of other lactobacilli in Trial II. A) Group-1, BV EcoVag®, B) Group-2, VVC EcoVag®, C) Group-3, VVC control (No EcoVag®). Table S3 Frequencies of isolation of other *Lactobacillus* species in Trial II following start of treatment until 6-month follow up. Table S4 Association between colonisation of vagina with lactobacilli and cure of BV and VVC (until 6-month follow up).
