# Supplementary figures and images for: Vaginal colonisation by probiotic lactobacilli and clinical outcome in women conventionally treated for bacterial vaginosis and yeast infection
Source: BMC Infect Dis. 2015 Jul 3;15:255. doi: 10.1186/s12879-015-0971-3 (PMC4489123; doi:10.1186/s12879-015-0971-3)

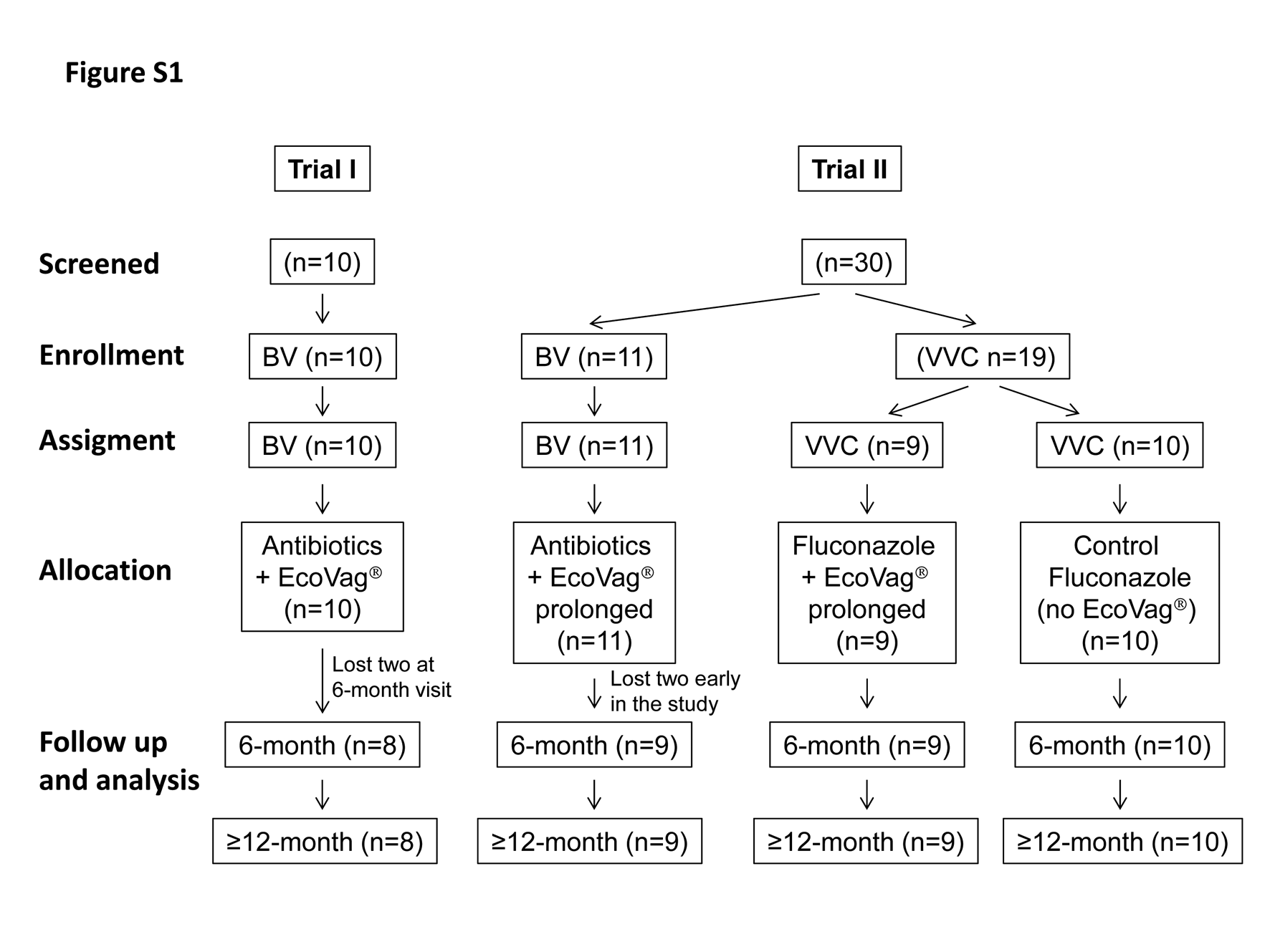

Supplement: Additional file 1: Figure S1. — Participants flow diagram. [file 12879_2015_971_MOESM1_ESM.tiff]

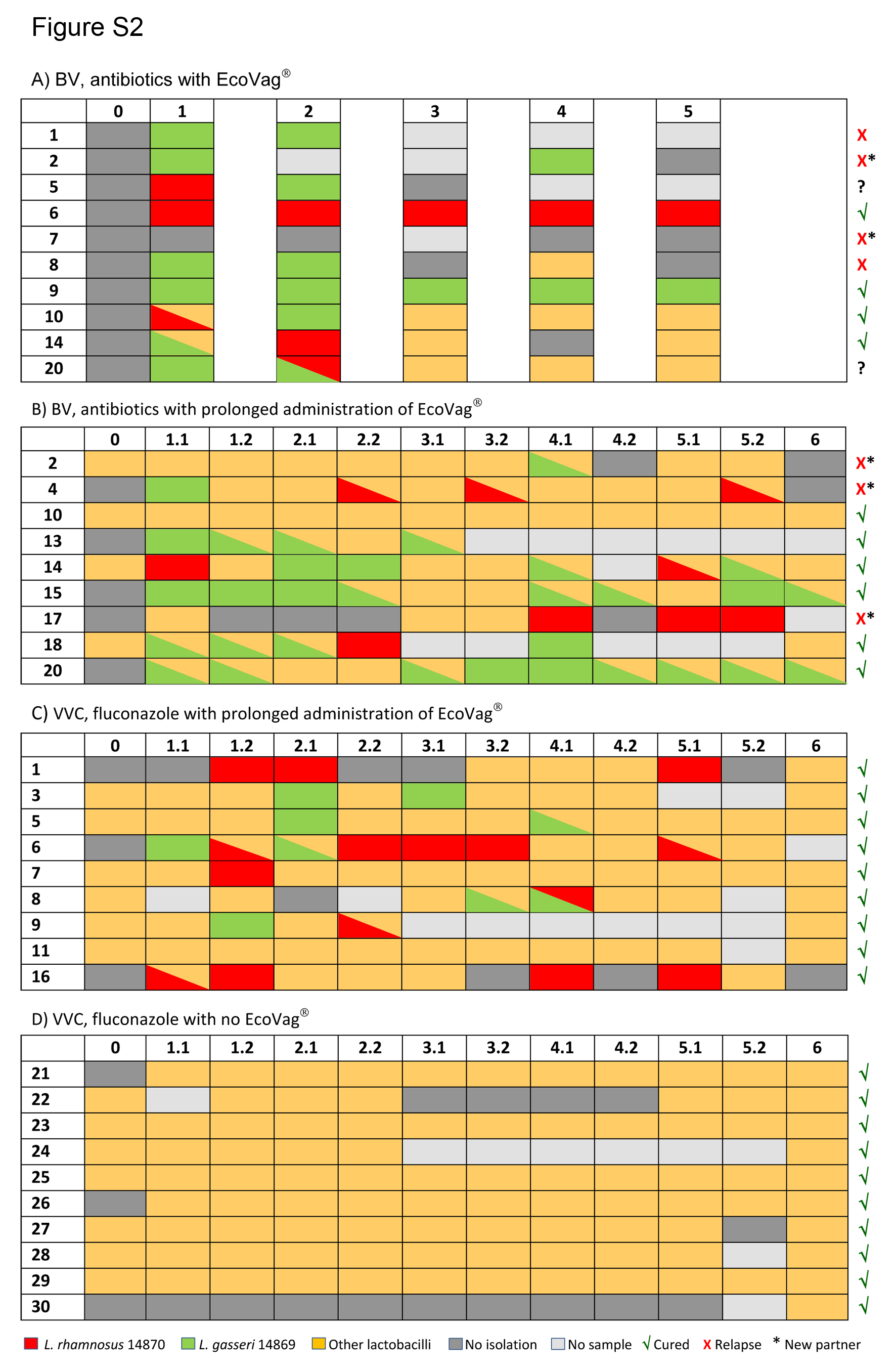

Supplement: Additional file 2: Figure S2. — Isolation of EcoVag® strains and other lactobacilli during the treatment. Some samples were not provided during the studies (No sample). Women with a new sexual partner (New partner), cured women (√) and relapse (X) at 6-month follow up are indicated. [file 12879_2015_971_MOESM2_ESM.tiff]
