# Supplementary material for: Vaginal colonisation by probiotic lactobacilli and clinical outcome in women conventionally treated for bacterial vaginosis and yeast infection
Source: BMC Infect Dis. 2015 Jul 3;15:255. doi: 10.1186/s12879-015-0971-3 (PMC4489123; doi:10.1186/s12879-015-0971-3)
Supplement: Additional file 3: Table S1, Table S2, Table S3 and Table S4. — Colonisation with lactobacilli: number of women colonised and frequency of isolation. Table S2 Identification of other lactobacilli in Trial II. A) Group-1, BV EcoVag®, B) Group-2, VVC EcoVag®, C) Group-3, VVC control (No EcoVag®). Table S3 Frequencies of isolation of other Lactobacillus species in Trial II following start of treatment until 6-month follow up. Table S4 Association between colonisation of vagina with lactobacilli and cure of BV and VVC (until 6-month follow up). [file 12879_2015_971_MOESM3_ESM.docx]

| **Trial** | **Group** | **Number of women** |  | **Colonisation and isolation frequency of EcoVag^®^ strains and other lactobacilli** | | |
| --- | --- | --- | --- | --- | --- | --- |
|  |  |  |  | | **Women colonised** | **Frequency of isolation (%)*** |
| **I** | **BV**  **EcoVag^®^** | 10 | *L. gasseri* DSM 14869 | | 8 | 17/42 (41 %) |
|  |  |  | *L. rhamnosus* DSM 14870 | | 5 | 9/42 (21 %) |
|  |  |  | Either of the Ecovag^®^ strains | | 9 | 24/42 (57 %) ^a^ |
|  |  |  | A lactobacilli | | 4 | 10/42 (24 %) ^a, g^ |
|  |  |  | Any lactobacilli (EcoVag^®^ or other lactobacilli) | | 9 | 33/42 (79 %) |
| **II** | **Group-1 BV EcoVag^®^** | 9 | *L. gasseri* DSM 14869 | | 7 | 32/86 (37 %) ^b, e^ |
|  |  |  | *L. rhamnosus* DSM 14870 | | 4 | 9/86 (10 %) ^b^ |
|  |  |  | Either of the Ecovag^®^ strains | | 8 | 41/86 (48 %) ^c, d^ |
|  |  |  | Other lactobacilli | | 9 | 63/86 (73 %) ^d, g^ |
|  |  |  | Any lactobacilli (EcoVag^®^ or other lactobacilli) | | 9 | 79/86 (92 %) |
|  | **Group-2 VVC EcoVag^®^** | 9 | *L. gasseri* DSM 14869 | | 5 | 10/86 (12 %) ^e^ |
|  |  |  | *L. rhamnosus* DSM 14870 | | 6 | 16/86 (19 %) |
|  |  |  | Either of the Ecovag^®^ strains | | 8 | 24/86 (28 %) ^c, f^ |
|  |  |  | Other lactobacilli | | 9 | 62/86 (72 %) ^f^ |
|  |  |  | Any lactobacilli (EcoVag^®^ or other lactobacilli) | | 9 | 78/86 (91 %) |
|  | **Group-3 VVC**  **Control (no EcoVag^®^)** | 10 | *L. gasseri* DSM 14869 | | - | - |
|  |  |  | *L. rhamnosus* DSM 14870 | | - | - |
|  |  |  | Either of the Ecovag^®^ strains | | - | - |
|  |  |  | Other lactobacilli | | 10 | 87/101 (86%) |
|  |  |  | Any lactobacilli (EcoVag^®^ or other lactobacilli) | | 10 | 87/101 (86%) |

**Table S1. Colonisation with lactobacilli: number of women colonized and frequency of isolation.**

* The frequency of isolation was determined as the number of samples positive for EcoVag^®^ strains or other lactobacilli on the total number of samples for each group. Values sharing the same letter are significantly different using Fisher’s exact test, ^a, d^ P<0.01,

^b, e, f, g^ P<0.001, ^c^ P<0.05.

**Table S2. Identification of other lactobacilli in Trial II.**

|  | **Sample** | | | | | | | | | | | | |
| --- | --- | --- | --- | --- | --- | --- | --- | --- | --- | --- | --- | --- | --- |
| **Woman** | **0** | **1.1** | **1.2** | **2.1** | **2.2** | **3.1** | **3.2** | **4.1** | **4.2** | **5.1** | **5.2** | **6**  **(6-months follow up)** | **7**  **(12-months follow up)** |
| **2** | *L. paracasei*  *L. salivarius* | *L. gasseri* | *L. salivarius*  *L. gasseri*  *L. reuteri* | *L. gasseri*  *L. reuteri* | *L. gasseri* | *L. rhamnosus* | *L. reuteri* | **14869**  *L. reuteri* | NI | *L. reuteri* | *L. gasseri*  *L. reuteri* | NI  **X** | NS  **X** |
| **4** | NI | **14869** | *L. fermentum* | *L. crispatus* | *L. crispatus* | *L. crispatus* | **14870**  *L. crispatus* | *L. crispatus* | *L. crispatus* | *L. crispatus* | **14870**  *L. crispatus* | NI  **X** | NS  **X** |
| **10** | *L. fermentum* | *L. gasseri* | *L. gasseri*  *L. rhamnosus* | *L. gasseri* | *L. gasseri* | *L. gasseri* | *L. gasseri* | *L. gasseri* | *L. gasseri* | *L. gasseri* | *L. gasseri* | *L. gasseri* | *L. gasseri* |
| **13** | NI | **14869** | **14869**  *L. gasseri* | **14869**  *L. plantarum* | *L. plantarum* | **14869**  *L. plantarum*  *L. gasseri* | NS | NS | NS | NS | NS | NS | NS |
| **14** | *L. acidophilus* | 14870 | *L. gasseri* | **14869** | **14869** | *L. gasseri*  *L. plantarum* | *L. acidophilus* | **14869^b^**  *L. plantarum* | NS | **14870**  *L. gasseri*  *L. plantarum* | **14869**  *L. acidophilus* | *L. plantarum* | *L. plantarum* |
| **15** | NI | **14869** | **14869** | **14869** | **14869**  *L. gasseri* | *L. gasseri*  *L. plantarum* | *L. gasseri* | **14869**  *L. gasseri* | **14869**  *L. gasseri* | **14869**  *L. acidophilus* | **14869** | **14869**  *L. gasseri* | *L. gasseri* |
| **17** | NI | *L. gasseri*  *L. rhamnosus* | NI | NI | NI | NI | *L. rhamnosus* | **14870** | NI | **14870** | **14870** | NS  **X** | NS  **X** |
| **18** | NI | **14869**  *L. fermentum* | **14869**  *L. fermentum* | **14869**  *L. fermentum* | **14870** | NS | NS | **14869** | NS | NS | NS | *L. iners* | *L. gasseri*  *L. crispatus* |
| **20** | NI | **14869**  *L. plantarum* | **14869**  *L. plantarum* | *L. gasseri* | *L. vaginalis*  *L. plantarum* | **14869**  *L. vaginalis* | **14869** | **14869** | **14869***. vaginalis* | **14869**  *L. vaginalis* | **14869**  *L. rhamnosus* | **14869**  *L. rhamnosus* | *L. gasseri* |

1. Group-1, BV EcoVag^®^

**14869**: *L. gasseri* DSM 14869

**14870**: *L. rhamnosus* DSM 14870

NI: No isolation

NS: No sample

**X**: Relapse at 6- and 12- months follow up

|  | Sample | | | | | | | | | | | | |
| --- | --- | --- | --- | --- | --- | --- | --- | --- | --- | --- | --- | --- | --- |
| **Woman** | **0** | **1.1** | **1.2** | **2.1** | **2.2** | **3.1** | **3.2** | **4.1** | **4.2** | **5.1** | **5.2** | **6**  **(6-months follow up)** | **7**  **(12-months follow up)** |
| **1** | NI | NI | **14870** | **14870** | NI | NI | *L. iners* | *L. iners* | *L. iners* | **14870** | NI | *L. iners* | *L. gasseri* |
| **3** | *L. gasseri*  *L. crispatus* | *L. gasseri* | *L. gasseri*  *L. paracasei* | **14869** | *L. gasseri* | **14869** | *L. gasseri*  *L. jensenii* | *L. gasseri* | *L. gasseri* | NS | NS | *L. gasseri* | *L. iners* |
| **5** | *L. plantarum* | *L. gasseri* | *L. gasseri*  *L. fermentum* | **14869** | *L. gasseri* | *L. gasseri* | *L. gasseri*  *L. plantarum* | **14869**  *L. gasseri* | *L. gasseri*  *L. plantarum* | *L. gasseri* | *L. gasseri* | *L. gasseri* | *L. gasseri*  *L. plantarum* |
| **6** | NI | **14869** | **14870**  *L. gasseri* | **14869**  *L. gasseri* | **14870** | **14870** | **14870**  **14869** | **14870**  *L. vaginalis* | *L. vaginalis* | **14870**  *L. vaginalis* | *L. vaginalis* | NS | NI |
| **7** | *L. crispatus*  *L. jensenii* | *L. crispatus* | **14870** | *L. crispatus* | *L. crispatus* | *L. crispatus* | *L. crispatus* | *L. crispatus*  *L. jenseneii* | *L. crispatus* | *L. crispatus* | *L. crispatus*  *L. vaginalis*  *L. jenseneii* | *L. vaginalis*  *L. jensenii* | *L. crispatus*  *L. gasseri* |
| **8** | *L. gasseri* | NS | *L. gasseri* | NI | NS | *L. rhamnosus* | **14869**  *L. gasseri* | **14869**  **14870** | *L. iners*  *L. coleohominis* | *L. gasseri* | NS | *L. iners* | *L. iners* |
| **9** | *L. gasseri* | *L. gasseri* | **14869** | *L. gasseri* | **14870**  *L. gasseri* | NS | NS | NS | NS | NS | NS | *L. gasseri* | *L. gasseri* |
| **11** | *L. crispatus* | *L. crispatus*  *L. reuteri* | *L. crispatus*  *L. paracasei* | *L. crispatus*  *L. plantarum* | *L. crispatus* | *L. crispatus* | *L. crispatus* | *L. crispatus*  *L. rhamnosus* | *L. paracasei* | *L. crispatus* | NS | *L. crispatus* | *L. crispatus* |
| **16** | NI | **14870**  *L. gasseri* | **14870** | *L. gasseri*  *L. reuteri* | *L. iners* | *L. rhamnosus* | NI | **14870** | NI | **14870** | *L. rhamnosus* | NI | NI |

1. Group-2, VVC EcoVag^®^

**14869**: *L. gasseri* DSM 14869

**14870**: *L. rhamnosus* DSM 14870

NI: No isolation

NS: No sample

|  | **Sample** | | | | | | | | | | | | |
| --- | --- | --- | --- | --- | --- | --- | --- | --- | --- | --- | --- | --- | --- |
| **Woman** | **0** | **1.1** | **1.2** | **2.1** | **2.2** | **3.1** | **3.2** | **4.1** | **4.2** | **5.1** | **5.2** | **6**  **(6-months follow up)** | **7**  **(12-months follow up)** |
| **21** | NI | *L. gasseri* | *L. gasseri* | *L. gasseri* | *L. gasseri*  *L. crispatus* | *L. gasseri*  *L. crispatus* | *L. gasseri* | *L. gasseri* | *L. gasseri* | *L. gasseri* | *L. gasseri* | *L. gasseri* | NS |
| **22** | *L. crispatus* | NS | *L. reuteri*  *L. paracasei* | *L. paracasei*  *L. reuteri* | *L. paracasei*  *L. reuteri* | NI | NI | NI | NI | *L. paracasei* | *L. jensenii* | *L. crispatus* | NS |
| **23** | *L. crispatus*  *L. jenseneii* | *L. crispatus* | *L. crispatus* | *L. crispatus*  *L. rhamnosus* | *L. crispatus*  *L. rhamnosus* | *L. crispatus*  *L. jensenii* | *L. crispatus* | *L. crispatus* | *L. crispatus* | *L. crispatus* | *L. crispatus* | *L. reuteri* | NS  **X** |
| **24** | *L. crispatus*  *L. vaginalis* | *L. paracasei* | *L. paracasei* | *L. crispatus* | *L. crispatus* | NS | NS | NS | NS | NS | NS | *L. crispatus*  *L. plantarum* | NS |
| **25** | *L. crispatus* | *L. crispatus*  *L. jensenii* | *L. crispatus* | *L. crispatus* | *L. crispatus* | *L. crispatus*  *L. jensenii* | *L. crispatus* | *L. crispatus*  *L. gasseri* | *L. crispatus* | *L. crispatus* | *L. crispatus* | *L. crispatus* | NS  **X** |
| **26** | NI | *L. crispatus* | *L. crispatus* | *L. crispatus* | *L. crispatus* | *L. crispatus* | *L. crispatus* | *L. crispatus* | *L. crispatus* | *L. crispatus* | NS | *L. iners* | NS |
| **27** | *L. gasseri* | *L. gasseri* | *L. gasseri* | *L. gasseri* | *L. gasseri* | *L. gasseri*  *L. crispatus* | *L. gasseri*  *L. crispatus* | *L. gasseri* | *L. gasseri* | *L. gasseri* | NS | *L. gasseri* | NS |
| **28** | *L. crispatus* | *L. crispatus* | *L. crispatus* | *L. crispatus* | *L. crispatus*  *L. gasseri* | *L. crispatus* | *L. crispatus* | *L. crispatus* | *L. crispatus* | *L. crispatus*  *L. reuteri* | NS | *L. fermentum* | NS  **X** |
| **29** | *L. fermentum* | *L. crispatus*  *L. fermentum* | *L. crispatus* | *L. gasseri*  *L. fermentum* | *L. crispatus*  *L. gasseri* | *L. gasseri* | *L. gasseri* | *L. gasseri*  *L. fermentum* | *L. gasseri*  *L. fermentum* | *L. gasseri* | *L. gasseri* | *L. reuteri* | NS |
| **30** | NI | NI | NI | NI | NI | NI | NI | NI | NI | NI | NS | *L. gasseri* | NS |

1. Group-3, VVC control (No EcoVag^®^)

**14869**: *L. gasseri* DSM 14869

**14870**: *L. rhamnosus* DSM 14870

NI: No isolation

NS: No sample

**X**: Relapse at 12-months follow up

**Table S3. Frequencies of isolation of other *Lactobacillus* species following start of treatment until 6-months follow up in Trial II.**

|  |  | **Number of samples positive for each *Lactobacillu*s species (%)** | | | | | | | | | | | |
| --- | --- | --- | --- | --- | --- | --- | --- | --- | --- | --- | --- | --- | --- |
|  | **Total samples** | ***L.***  ***gasseri*** | ***L.***  ***crispatus*** | ***L.***  ***jensenii*** | ***L.***  ***iners*** | ***L.***  ***vaginalis*** | ***L.***  ***reuteri*** | ***L. paracasei*** | ***L. plantarum*** | ***L. rhamnosus*** | ***L. fermentum*** | ***L. acidophilus*** | ***L. salivarius*** |
| **Group-1**  **BV EcoVag^®^** | 86 | 29 (33.7) | 8 (9.3) ^a^ | 0 (0) | 1 (1.2) | 4 (4.7) | 6 (7.0) | 0 (0.0) | 12 (14.0) | 6 (7.0) | 4 (4.7) | 3 (3.5) | 1 (1.2) |
| **Group-2 VVC EcoVag^®^** | 86 | 28 (32.5) | 18 (20.9) | 2 (2.3) | 7 (8.1) | 6 (7.0) | 2 (2.3) | 3 (3.5) | 3 (3.5) | 4 (4.7) | 1 (1.2) | 0 (0) | 0 (0) |
| **Group-3 VVC**  **control** | 101 | 31 (30.6) | 50 (49.5) | 0 (0.0) | 1 (1.0) | 0 (0.0) | 6 (5.9) | 6 (5.9) | 1 (1.0) | 2 (2.0) | 5 (5.0) | 0 (0) | 0 (0) |

^a^ Significantly lower proportion of samples positive for *L. crispatus* in Group-1 (BV EcoVag^®^) compared to Group-2 (VVC EcoVag^®^)(P<0.05) and Group-3 (P<0.01).

| **Trial** | **Group** | **Number of women** | **Number of women**  **(6-month)**  **Cured/**  **relapse** | **Isolation frequency and colonisation by EcoVag^®^ strains and other lactobacilli** | | |
| --- | --- | --- | --- | --- | --- | --- |
|  |  |  |  |  | **Women colonised** | **Frequency of isolation (%)*** |
| I | BV  EcoVag^®^ | 8 |  | *L. gasseri* DSM 14869 | 3 | 8/20 (40 %) |
|  |  |  |  | *L. rhamnosus* DSM 14870 | 3 | 7/20 (35 %) |
|  |  |  | Cured | Either of the Ecovag^®^ strains | 4 | 14/20 (70 %) |
|  |  |  | 4 | Other lactobacilli | 2 | 6/20 (30 %) |
|  |  |  |  | Any lactobacilli | 4 | 19/20 (95 %) ^a^ |
|  |  |  | Relapse  4 | *L. gasseri* DSM 14869 | 3 | 6/14 (43 %) |
|  |  |  |  | *L. rhamnosus* DSM 14870 | 0 | 0/14 (0 %) |
|  |  |  |  | Either of the Ecovag^®^ strains | 3 | 6/14 (43 %) |
|  |  |  |  | Other lactobacilli | 1 | 1/14 (7 %) |
|  |  |  |  | Any lactobacilli | 3 | 7/14 (50 %) ^a^ |
| II | BV  EcoVag^®^ |  | Cured  6 | *L. gasseri* DSM 14869 | 5 | 30/54 (56 %) ^b^ |
|  |  |  |  | *L. rhamnosus* DSM 14870 | 2 | 3/54 (6 %) |
|  |  |  |  | Either of the Ecovag^®^ strains | 5 | 33/54 (61 %) ^c^ |
|  |  | 9 |  | Other lactobacilli | 6 | 42/54 (78 %) |
|  |  |  |  | Any lactobacilli | 6 | 54/54 (100 %) ^d^ |
|  |  |  |  | *L. gasseri* DSM 14869 | 2 | 2/32 (6 %) ^b^ |
|  |  |  |  | *L. rhamnosus* DSM 14870 | 2 | 6/32 19 %) |
|  |  |  | Relapse | Either of the Ecovag^®^ strains | 3 | 8/32 (25 %) ^c^ |
|  |  |  | 3 | Other lactobacilli | 3 | 21/32 (66 %) |
|  |  |  |  | Any lactobacilli | 3 | 25/32 (78 %) ^d^ |

**Table S4. Association between vaginal colonisation by lactobacilli and cure of BV and VVC (until 6-month follow up).**

* The frequency of isolation was determined as the number of samples positive for EcoVag^®^ strains or other lactobacilli on the total number of samples for each group. Values sharing the same letter are significantly different using Fisher’s exact test, P<0.01 ^a, c^, P<0.001 ^b, d^.

| **Trial** | **Group** | **Number of women** | **Number of women**  **(6-month)**  **Cured/**  **relapse** | **Isolation frequency and colonisation by EcoVag^®^ strains and other lactobacilli** | | |
| --- | --- | --- | --- | --- | --- | --- |
|  |  |  |  |  | **Women colonised** | **Frequency of isolation (%)*** |
| II | VVC  EcoVag^®^ | 9 |  | *L. gasseri* DSM 14869 | 5 | 10/86 (12 %) |
|  |  |  |  | *L. rhamnosus* DSM 14870 | 6 | 16/86 (19 %) |
|  |  |  | Cured | Either of the Ecovag^®^ strains | 8 | 24/86 (28 %) |
|  |  |  | 9 | Other lactobacilli | 9 | 62/86 (72 %) |
|  |  |  |  | Any lactobacilli | 9 | 78/86 (91 %) |
|  |  |  | Relapse  0 | *L. gasseri* DSM 14869 | - | - |
|  |  |  |  | *L. rhamnosus* DSM 14870 | - | - |
|  |  |  |  | Either of the Ecovag^®^ strains | - | - |
|  |  |  |  | Other lactobacilli | - | - |
|  |  |  |  | Any lactobacilli | - | - |
|  | VVC  Control (no EcoVag^®^) | 10 | Cured  10 | *L. gasseri* DSM 14869 | - | - |
|  |  |  |  | *L. rhamnosus* DSM 14870 | - | - |
|  |  |  |  | Either of the Ecovag^®^ strains | - | - |
| II |  |  |  | Other lactobacilli | 10 | 87/101 (86%) |
|  |  |  |  | Any lactobacilli | 10 | 87/101 (86%) |
|  |  |  | Relapse  0 | *L. gasseri* DSM 14869 | - | - |
|  |  |  |  | *L. rhamnosus* DSM 14870 | - | - |
|  |  |  |  | Either of the Ecovag^®^ strains | - |  |
|  |  |  |  | Other lactobacilli | - | - |
|  |  |  |  | Any lactobacilli | - | - |

* The frequency of isolation was determined as the percentage of samples positive for EcoVag^®^ strains or other lactobacilli on the total number of samples for each group (%). Values sharing the same letter are significantly different using Fisher’s exact test, P<0.01 ^a, c^, P<0.001 ^b, d^.
